# Supplementary figures and images for: Diverse mating phenotypes impact the spread of wtf meiotic drivers in Schizosaccharomyces pombe
Source: eLife. 2021 Dec 13;10:e70812. doi: 10.7554/eLife.70812 (PMC8789285; doi:10.7554/eLife.70812)

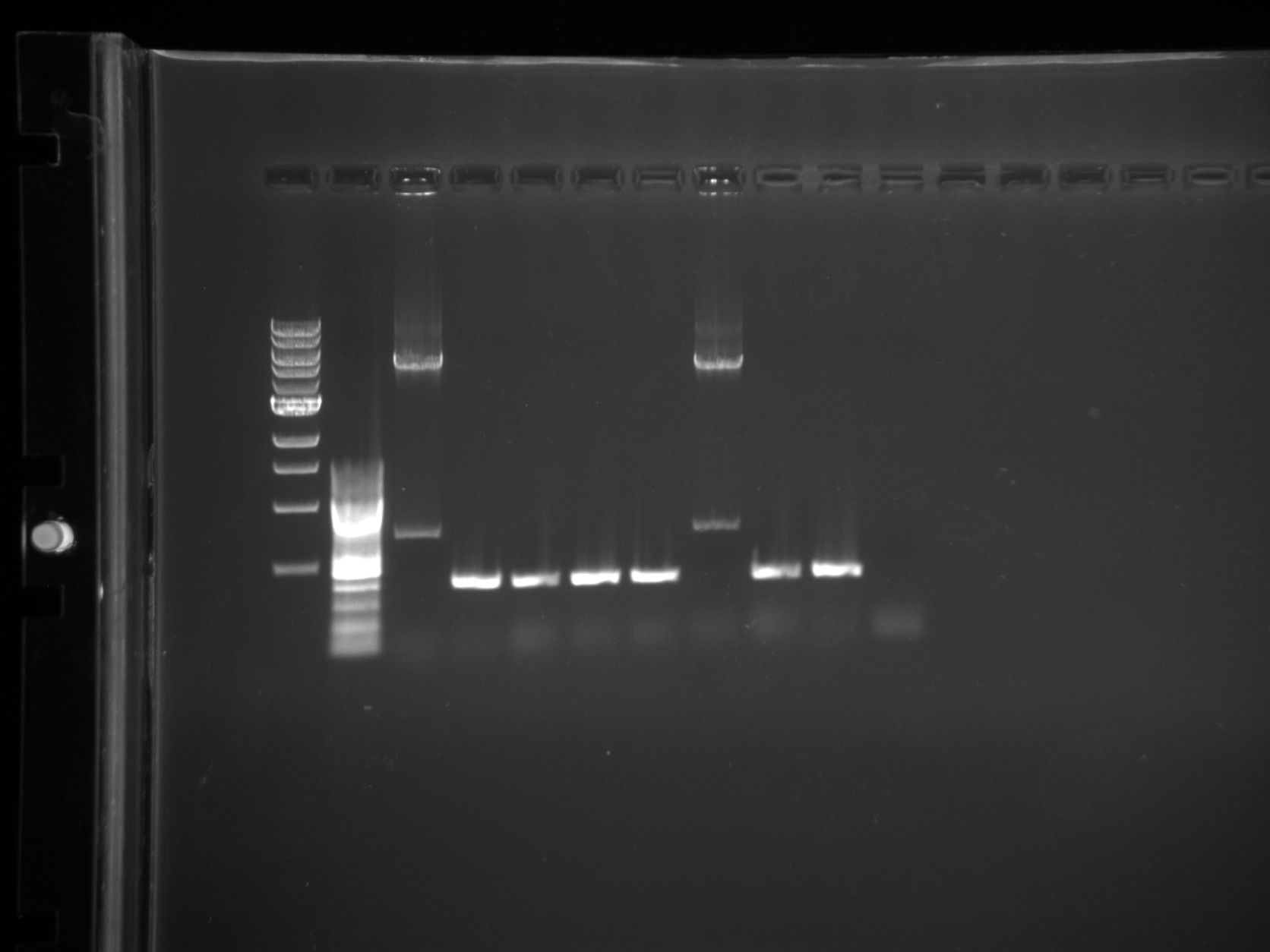

Supplement: Figure 2—figure supplement 1—source data 1. [file elife-70812-fig2-figsupp1-data1.pdf]

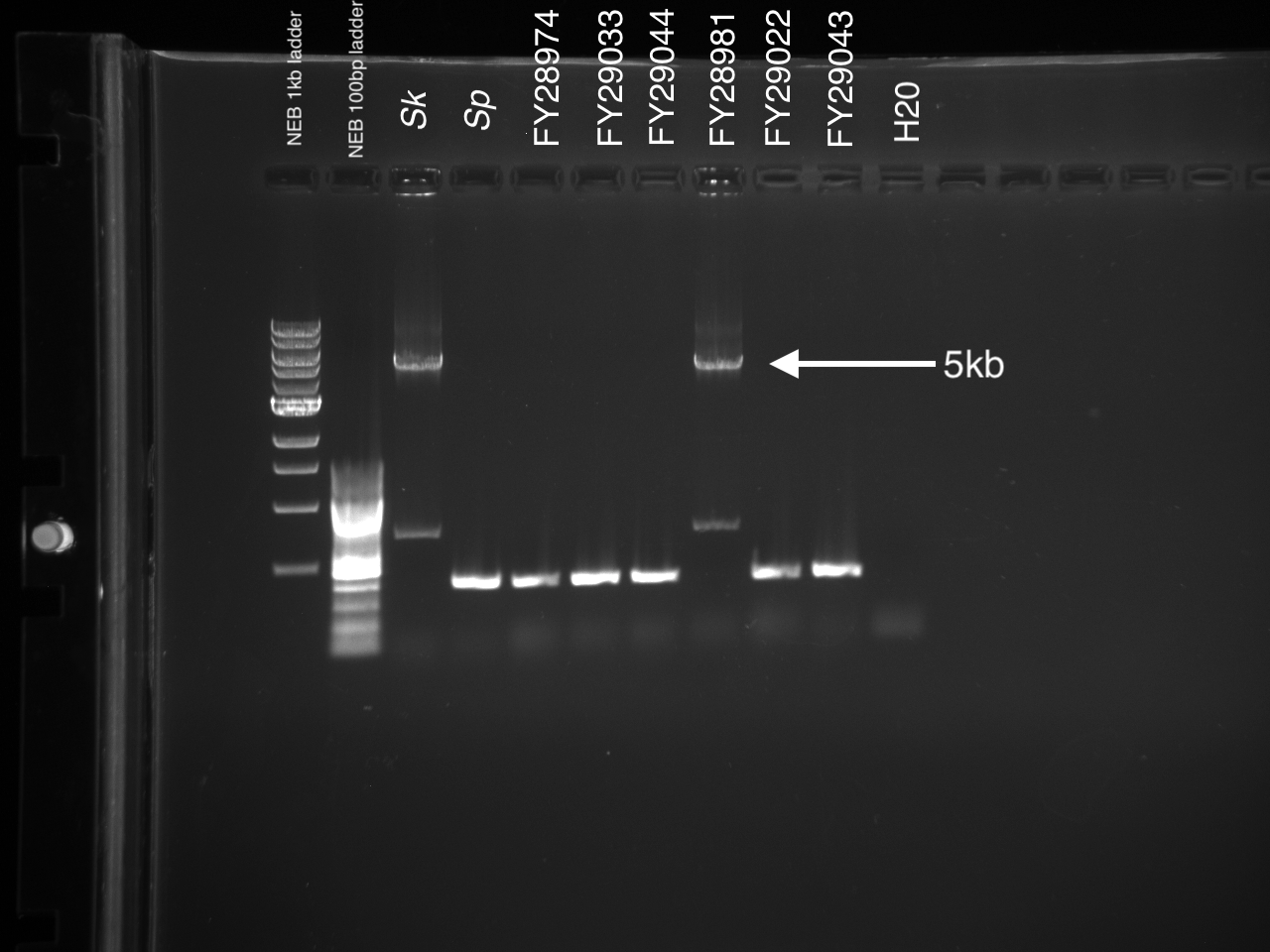

Supplement: Figure 2—figure supplement 1—source data 2. [file elife-70812-fig2-figsupp1-data2.tiff]
